# Medication errors in community pharmacies: Evaluation of a standardized safety program

**DOI:** 10.1016/j.rcsop.2022.100218

**Published:** 2022-12-21

**Authors:** Shaleesa Ledlie, Tara Gomes, Lisa Dolovich, Chantelle Bailey, Saira Lallani, Delia Sinclair Frigault, Mina Tadrous

**Affiliations:** a Li Ka Shing Knowledge Institute, Unity Health, Toronto, Ontario, Canada; b Ontario Drug Policy Research Network, Toronto, Ontario, Canada; c Leslie Dan Faculty of Pharmacy, University of Toronto, Toronto, Ontario, Canada; d Institute for Health Policy, Management, and Evaluation, University of Toronto, Toronto, Ontario, Canada; e ICES, Toronto, Ontario, Canada; f Women's College Research Institute, Toronto, Ontario, Canada; g Ontario College of Pharmacists, Toronto, Ontario, Canada

**Keywords:** Medication errors, Community pharmacy, Pharmacists, Pharmacy staff, AIMS, Assurance and Improvement in Medication Safety, OCP, Ontario College of Pharmacists

## Abstract

**Background:**

The mandated reporting of medication-related errors in community pharmacies including incidents resulting in inappropriate medication use and near misses intercepted before reaching the patient can be utilized as learning opportunities to aid in the prevention of future events.

**Objectives:**

To examine reporting uptake, trends, and initial learnings from medication errors reported by community pharmacists to the Assurance and Improvement in Medication Safety (AIMS) Program based in Ontario, Canada between April 1st, 2018, and June 30th, 2021.

**Methods:**

A descriptive analysis was conducted of all events reported to the AIMS Program during the study period. The web-based reporting form includes a series of mandatory and optional fields completed by the reporter. Individual medications were grouped into broader classes prior to conducting the analysis.

**Results:**

Among the 31,768 event reports received from 2856 community pharmacies, there were 19,639 incidents and 12,129 near misses. Low reporting followed by a rapid increase was observed during expansion of the AIMS Program in 2018, with almost 60% of Ontario community pharmacies submitting at least 1 event over the study period. In most cases (90.5%), no patient harm was reported. The most frequent event types involved the incorrect drug (19.5%), concentration (17.2%) or quantity (14.5%). Approximately 25% of events were identified by the involved patient or their agent. When looking at medication classes, antihypertensives, opioids and antidepressants were involved in over one-quarter of overall and higher severity events. Environmental staffing problems and interruptions were the contributory factor and sub-factor most frequently reported, respectively.

**Conclusions:**

This study provides insights into engagement with the AIMS Program by Ontario community pharmacy teams since implementation in 2018. The identification of the circumstances and medications associated with both incidents and near misses, aids in the continued development of strategies and processes to help prevent future events.

## Highlights

• Community pharmacy engagement with the AIMS Program has grown since implementation.
• Commonly reported events involved the incorrect drug, concentration, or quantity.
• In most cases no patient harm was reported.
• Reporting by pharmacy teams will help develop strategies to prevent future events.

## 1 Introduction

Medication errors are preventable events that may cause inappropriate medication use or patient harm.^1^ Across North America, approximately one-quarter of all medication errors with resulting patient harm occur in community pharmacies.^2^^,^^3^ Common medication errors involve dispensing of the incorrect drug, concentration, or quantity due to various environmental and drug-related factors including prescription errors, staffing shortages and medications with look-alike names or packaging.^4^^,^^5^ The annual cost of medication errors in Canada is estimated to be $2.6 billion per year, with additional costs incurred due to lost productivity and time away from work.6., 7, 8 To improve patient safety worldwide, there have been calls for an improved culture of transparency surrounding medication errors and the strategies implemented by healthcare institutions to aid in the reduction of recurrent events.^2^^,^^11^^,^^12^ There are also global initiatives targeted at reducing preventable medication errors, such as the World Health Organization's *Medication Without Harm* safety challenge. This program aims to reduce avoidable patient harms related to medications across all health systems by 50% between 2017 and 2022.^9^ Further, the Institute for Safe Medication Practices retrospectively collects reports related to medication errors from healthcare institutions across Canada with the goal of identifying common errors prior to occurrence and implementing effective preventative strategies.^10^

The Ontario College of Pharmacists (OCP) is the registering and regulatory body for the profession of pharmacy in Ontario, Canada's largest province.^13^ In 2018, the OCP initiated the Assurance and Improvement in Medication Safety (AIMS) Program with the goal of reducing the risk of patient harm caused by medication errors in community pharmacies. There are approximately 4700 community pharmacies located across Ontario, which provide retail pharmacy services and care to the public^14^^,^^15^ Following a successful pilot program at 100 community pharmacies, the AIMS Program was expanded provincially beginning in November 2018.^16^ All Ontario community pharmacy professionals are mandated to follow set requirements which include reporting, documenting, analyzing, and sharing learnings from medication-related events.^17^ The utilization of the web-based AIMS Pharmapod® platform allows all community pharmacies to meet this requirement while standardizing the reporting process. The anonymized reporting of both medication incidents and near misses is required. Incidents are defined as preventable events that may cause inappropriate medication use or patient harm and near misses as events that could have led to inappropriate medication use or patient harm but were intercepted before reaching the patient.^1^

There is currently limited information on the rate at which medication errors occur within community pharmacies across Canada and the United States, with large heterogeneity in the volume of reports captured through differing reporting systems.^18^ Evidence suggests that the development of a confidential, mandated medication error reporting system may lead to increased reporting and the ability to detect rarer events and emerging problems across healthcare settings.^19^, ^20^, ^21^ Therefore, we sought to evaluate the uptake in reporting of medication-related events since the launch of the AIMS Program in 2018, describe reporting trends, and identify initial learnings that can be used to help develop strategies to prevent future medication errors in community pharmacies.

## 2 Methods

### 2.1 Study design

A descriptive analysis was conducted of all medication incidents and near misses reported by community pharmacies through the AIMS Pharmapod platform between April 1st, 2018, and June 30th, 2021. All reports originated from community pharmacies located across Ontario. This study is reported as per the Strengthening the Reporting of Observational Studies in Epidemiology (STROBE) guidelines,^22^ and was approved by the Unity Health Toronto Research Ethics Board (No. 21–196).

#### 2.1.1 Data source

Anonymized reports made through the AIMS Pharmapod platform are captured and stored in a secured database. Pharmacy professionals have access to the platform and must complete several mandatory eLearning modules focused on the AIMS Program objectives, importance and how to complete the electronic report forms.^23^ Each standardized form contains a series of both mandatory and optional fields for completion, listed in Appendix A. Personal health information on the involved patient is not collected except for month and year of birth and gender. Event severity is only collected for incidents and based on an assessment made by the pharmacy professional at the time of reporting, categorized as: none, mild (i.e., symptoms were mild in nature, temporary and short term), moderate (i.e., symptoms resulted in hospitalization or permanent harm), severe (i.e., symptoms resulted in major permanent long-term loss of functions or harm), and death (i.e., reason to believe the event caused or hastened the patient's death). Following an evaluation process, the pharmacy professional involved in each event is instructed to include all factors and associated sub-factors that they believe contributed to the event's occurrence, and as such >1 option can be selected. Once a form is initiated, additional details can be added over several consecutive sessions.

#### 2.1.2 Data analysis

All reported events during the study period were included in the analysis, as documented in the AIMS Pharmapod database. There were no exclusion criteria applied. Descriptive statistics were used to describe quarterly events, the number of reporting pharmacies, report characteristics, and the medication classes most frequently identified overall and in higher severity events. All data were summarized using counts and proportions stratified by the event type (i.e., incident or near miss), as relevant. Missing data were summarized as a separate category. A Lorenz curve was created to assess the cumulative distribution of reported events among community pharmacies. The corresponding Gini coefficient was calculated, which ranges from values of 0 to 1 representing perfect equality and extreme inequality in reporting, respectively.^24^

Patient age was calculated based on the recorded month and year of birth, rounded to the nearest full year, and categorized into 5 groups (<25, 25–44, 45–64, ≥65 years and unknown). All medication names were grouped into broader classes (e.g., antihypertensives, antidepressants) based on the review of 2 researchers (M.T and S.L) using the Anatomical Therapeutic Chemical (ATC) classification system, who discussed and reached an agreement on any discrepancies. When assessing the top medication classes involved in medication-related events, the medications dispensed rather than prescribed were utilized, to capture the medications received or intercepted prior to receipt by patients. For reports involving >1 medication, all medications were considered independently. All analyses were conducted using SAS version 9.4 (SAS institute, Cary, North Carolina, USA).

## 3 Results

**Fig. 1:**
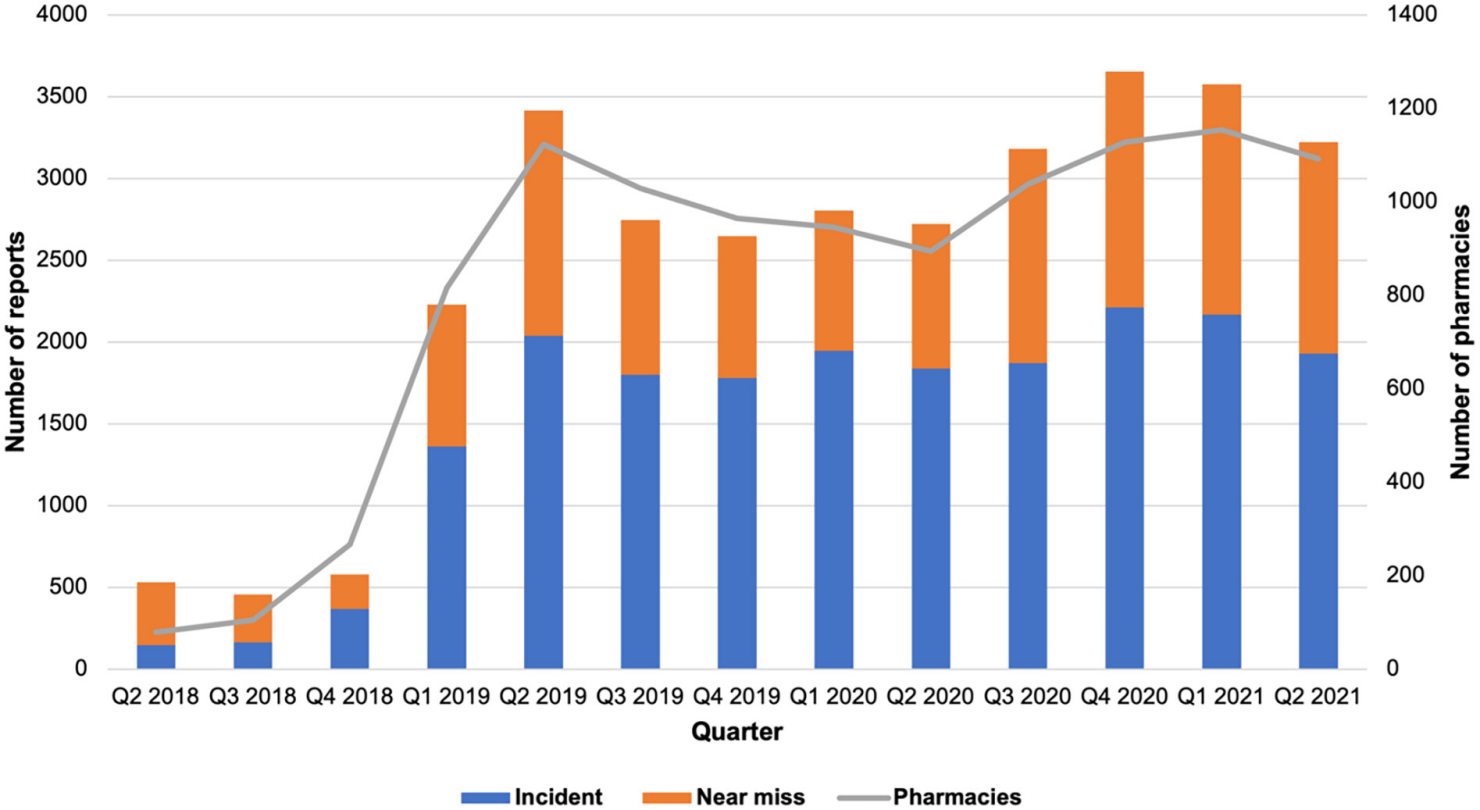
Quarterly reporting by pharmacies, overall and stratified by incidents and near misses from April 2018 to March 2021.

Between April 1st, 2018, and June 30th, 2021, there were 31,768 events captured in the AIMS Pharmapod platform (19,639 [61.8%] incidents and 12,129 [38.2%] near misses). Reports were received from 2856 unique community pharmacies, representing approximately 60% of community pharmacies in Ontario. The total number of incidents, near misses and reporting pharmacies, summarized on a quarterly basis can be seen in Fig. 1. Low reporting was observed during initial years of the program prior to expansion to all Ontario community pharmacies beginning at the end of 2018. Following this, a sharp uptake in reporting occurred during the first quarter of 2019 with relatively stable quarterly reporting observed during the remainder of the study period. The number of reporting pharmacies peaked in the first quarter of 2021 (*N* = 1154), representing almost 25% of Ontario community pharmacies. The total number of reports followed a similar pattern, reaching a peak in the fourth quarter of 2020 (*N* = 3654). After the initial stabilization period in 2018, approximately two-thirds of all events were characterized as incidents each quarter (range: 59% to 69%).

Patients who were ≥ 65 years of age (35.3%) and those identified as male (57.2%) were more frequently involved in events (Table 1). The event types most reported across the study period included the incorrect drug (19.5%), concentration (17.2%) or quantity (14.5%). Most events did not result in any patient harm (90.5%), however of those that did impose harm, the majority were classified as mild (4.9%), with moderate (0.9%) and severe (0.1%) events, and those resulting in death (0.1%) occurring rarely. Almost three quarters of all events occurred either during order entry (39.5%) or medication dispensing (33.6%) and were frequently identified by either a pharmacist (26.6%) or the involved patient (20.0%). The median number of contributory factors and sub-factors selected per report was 1 with an interquartile range of 0. The contributory factors most identified included environmental staffing problems (29.9%), and a lack of quality control systems (22.6%) or staff education (14.4%). Similarly, the top sub-factors overall were interruptions (12.9%), performing an independent double-check for high alert medications (12.8%) and high-volume dispensing (9.4%).

**Table 1:** Characteristics of reported events from Ontario community pharmacies, overall and stratified by incidents and near misses.

Characteristic	Reported events		
	Overall (*N* = 31,768)	Incident (*N* = 19,639)	Near Miss (*N* = 12,129)
Patient age			
0–24	6935 (21.8%)	4362 (22.2%)	2573 (21.2%)
25–44	6084 (19.2%)	3747 (19.1%)	2337 (19.3%)
45–64	6145 (19.3%)	3714 (18.9%)	2431 (20.0%)
65+	11,201 (35.3%)	6905 (35.2%)	4296 (35.4%)
Missing	1403 (4.4%)	911 (4.6%)	492 (4.1%)
Patient gender			
Male	18,176 (57.2%)	11,318 (57.6%)	6858 (56.5%)
Female	13,275 (41.8%)	8124 (41.4%)	5151 (42.5%)
Other	271 (0.9%)	168 (0.9%)	103 (0.8%)
Missing	46 (0.1%)	29 (0.1%)	17 (0.1%)
Event category			
Incorrect drug	6188 (19.5%)	4114 (20.9%)	2074 (17.1%)
Incorrect concentration	5470 (17.2%)	3614 (18.4%)	1856 (15.3%)
Incorrect quantity	4594 (14.5%)	2418 (12.3%)	2176 (17.9%)
Incorrect patient	2484 (7.8%)	1481 (7.5%)	1003 (8.3%)
Medication incorrect label	1695 (5.3%)	763 (3.9%)	932 (7.7%)
Incorrect frequency	1609 (5.1%)	894 (4.6%)	715 (5.9%)
Medication omitted medication dose	1308 (4.1%)	780 (4.0%)	528 (4.4%)
Incorrect dosage form	1107 (3.5%)	747 (3.8%)	360 (3.0%)
Incorrect duration	493 (1.6%)	245 (1.2%)	248 (2.0%)
Duplication of therapy	436 (1.4%)	290 (1.5%)	146 (1.2%)
Medication incorrect prescriber	391 (1.2%)	139 (0.7%)	252 (2.1%)
Passed expiry date	310 (1.0%)	118 (0.6%)	192 (1.6%)
Incorrect storage	310 (1.0%)	76 (0.4%)	234 (1.9%)
Professional services incident	256 (0.8%)	233 (1.2%)	23 (0.2%)
Prescribing error	170 (0.5%)	74 (0.4%)	96 (0.8%)
Medication inappropriately discontinued	163 (0.5%)	120 (0.6%)	43 (0.4%)
Drug therapy monitoring problem	157 (0.5%)	39 (0.2%)	118 (1.0%)
Incorrect route administration	119 (0.4%)	49 (0.2%)	70 (0.6%)
Other	4402 (13.9%)	3365 (17.1%)	1037 (8.5%)
Missing	106 (0.3%)	80 (0.4%)	26 (0.2%)
Severity			
None/Near miss	28,742 (90.5%)	16,613 (84.6%)	12,129 (100.0%)
Mild	1562 (4.9%)	1562 (8.0%)	0 (0.0%)
Moderate	278 (0.9%)	278 (1.4%)	0 (0.0%)
Severe	47 (0.1%)	47 (0.2%)	0 (0.0%)
Death	17 (0.1%)	17 (0.1%)	0 (0.0%)
Unknown	1122 (3.5%)	1122 (5.7%)	0 (0.0%)
Event stage			
Order entry	12,555 (39.5%)	7053 (35.9%)	5502 (45.4%)
Dispensing	10,669 (33.6%)	6533 (33.3%)	4136 (34.1%)
Product selection	3100 (9.8%)	1964 (10.0%)	1136 (9.4%)
Delivery	1298 (4.1%)	1073 (5.5%)	225 (1.9%)
Prescribing	943 (3.0%)	584 (3.0%)	359 (3.0%)
Communication	679 (2.1%)	526 (2.7%)	153 (1.3%)
Administration	473 (1.5%)	414 (2.1%)	59 (0.5%)
Storage	309 (1.0%)	179 (0.9%)	130 (1.1%)
Supply	306 (1.0%)	211 (1.1%)	95 (0.8%)
Other	1324 (4.2%)	1018 (5.2%)	306 (2.5%)
Missing	112 (0.4%)	84 (0.4%)	28 (0.2%)
Identified by			
Pharmacist	8459 (26.6%)	7001 (35.6%)	1458 (12.0%)
Patient	6339 (20.0%)	6339 (32.3%)	0 (0.0%)
Patient agent	1391 (4.4%)	1391 (7.1%)	0 (0.0%)
Pharmacy assistant	1260 (4.0%)	880 (4.5%)	380 (3.1%)
Pharmacy technician	1004 (3.2%)	609 (3.1%)	395 (3.3%)
Prescriber	878 (2.8%)	852 (4.3%)	26 (0.2%)
Pharmacy student	815 (2.6%)	410 (2.1%)	405 (3.4%)
Nurse	721 (2.3%)	709 (3.6%)	12 (0.1%)
Social worker	95 (0.3%)	69 (0.4%)	26 (0.2%)
Other HCP	38 (0.1%)	38 (0.2%)	0 (0.0%)
Missing	10,768 (33.9%)	1341 (6.8%)	9427 (77.7%)
Contributory factors⁎			
Environmental staffing problem	9487 (29.9%)	6294 (32.0%)	3193 (26.3%)
Lack of quality control systems	7195 (22.6%)	4696 (23.9%)	2499 (20.6%)
Lack of staff education	4578 (14.4%)	2598 (13.2%)	1980 (16.3%)
Miscommunication of drug order	2945 (9.3%)	2112 (10.8%)	833 (6.9%)
Drug-related issues	1546 (4.9%)	1057 (5.4%)	489 (4.0%)
Patient information missing	529 (1.7%)	367 (1.9%)	162 (1.3%)
Patient caregiver education problem	325 (1.0%)	295 (1.5%)	30 (0.2%)
Patient education problem	231 (0.7%)	192 (1.0%)	39 (0.3%)
Other	13,153 (41.4%)	8353 (42.5%)	4800 (39.6%)
Missing	113 (0.4%)	86 (0.4%)	27 (0.2%)
Contributory sub-factors†			
Interruptions	4086 (12.9%)	4019 (20.5%)	1214 (10.0%)
Independent check due to high-risk drugs	4053 (12.8%)	2445 (12.4%)	1608 (13.3%)
High volume dispensing	2995 (9.4%)	2053 (10.5%)	942 (7.8%)
Heavy workload	2982 (9.4%)	2138 (10.9%)	844 (7.0%)
Failure to follow established process	2742 (8.6%)	1427 (7.3%)	1315 (10.8%)
Misunderstood orders	1713 (5.4%)	1222 (6.2%)	491 (4.0%)
Fatigue	1531 (4.8%)	962 (4.9%)	569 (4.7%)
Equipment control checks	1440 (4.5%)	894 (4.6%)	546 (4.5%)
Noise	627 (2.0%)	447 (2.3%)	180 (1.5%)
New unfamiliar drug	538 (1.7%)	320 (1.6%)	218 (1.8%)

⁎ In some cases, multiple factors were selected per event, therefore percentages do not add up to 100.0%.

† Represents the top 10 most frequently selected contributory sub-factors.

**Fig. 2:**
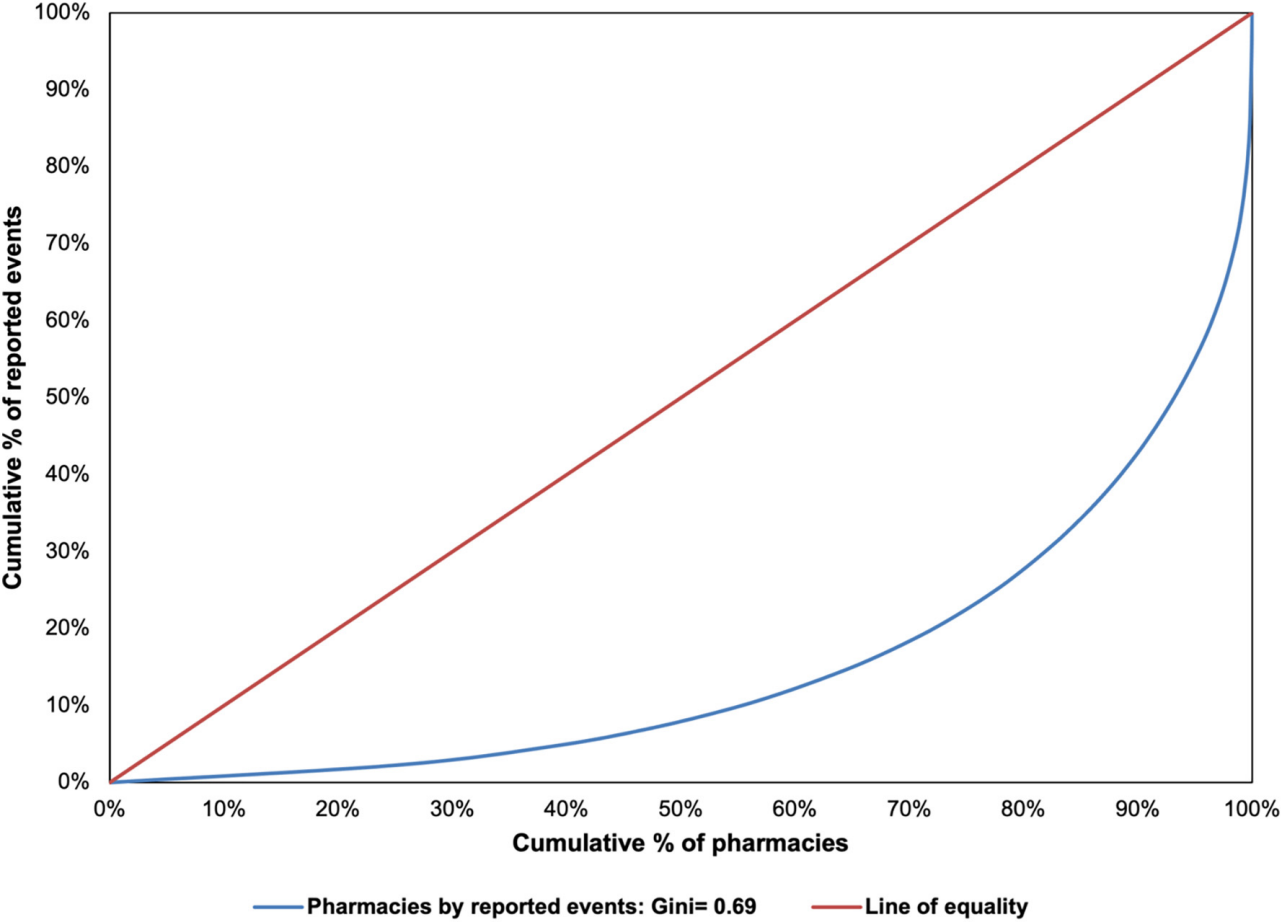
Lorenz curve: pharmacies by reported events.

Dispensed medications classified as antihypertensives (11.6%), opioids (7.5%), antidepressants (6.8%), and antibiotics (4.6%) were involved in the highest proportion of events (Table 2). When looking at higher severity events defined as those classified as moderate, severe, or resulting in death, opioids (13.7%), and antihypertensives (9.9%) were most frequently involved (Table 3). Opioids and antihypertensives contributed to 10.6% and 12.8% of severe events, and 52.9% and 11.8% of events resulting in death, respectively. Finally, the results from the Lorenz curve and corresponding Gini coefficient of 0.69 show that there is a relatively high degree of inequality in reporting among pharmacies (Fig. 2), with the number of events reported per pharmacy ranging from 1 to 1280. Among the 2856 community pharmacies included in the analysis, one-quarter reported only 1 event over the study period, and the top 10% highest reporting pharmacies were responsible for reporting 57.2% of events.

**Table 2:** Top twenty medication classes dispensed involved in events, stratified by incidents and near misses.

Medication class	Event type		
	Overall (N = 31,768)	Incident (N = 19,639)	Near miss (N = 12,129)
Antihypertensives	3688 (11.6%)	2348 (12.0%)	1340 (11.0%)
Opioids	2380 (7.5%)	1589 (8.1%)	791 (6.5%)
Antidepressants	2158 (6.8%)	1426 (7.3%)	732 (6.0%)
Antibiotics	1452 (4.6%)	825 (4.2%)	627 (5.2%)
Antidiabetics	1302 (4.1%)	923 (4.7%)	379 (3.1%)
Antipsychotics	1045 (3.3%)	549 (2.8%)	496 (4.1%)
Anticonvulsants	923 (2.9%)	591 (3.0%)	332 (2.7%)
Benzodiazepines	841 (2.6%)	519 (2.6%)	322 (2.7%)
Corticosteroids	753 (2.4%)	446 (2.3%)	307 (2.5%)
Proton-pump inhibitors (PPI)	758 (2.4%)	484 (2.5%)	274 (2.3%)
Antithyroids	664 (2.1%)	479 (2.4%)	185 (1.5%)
Vitamin and Minerals	643 (2.0%)	353 (1.8%)	290 (2.4%)
Anticoagulants	601 (1.9%)	367 (1.9%)	234 (1.9%)
Topicals and Dermatologics	582 (1.8%)	329 (1.7%)	253 (2.1%)
Antilipidemics	546 (1.7%)	359 (1.8%)	187 (1.5%)
Immunosuppressants	525 (1.7%)	264 (1.3%)	261 (2.2%)
Eye treatments	515 (1.6%)	327 (1.7%)	188 (1.6%)
Non-steroidal anti-inflammatory drugs (NSAID)	491 (1.5%)	292 (1.5%)	199 (1.6%)
Vaccines	486 (1.5%)	415 (2.1%)	71 (0.6%)
Statins	460 (1.4%)	302 (1.5%)	158 (1.3%)

**Table 3:** Top twenty medication classes dispensed involved in higher severity events.

Medication class	Harm level			
	Overall (*N* = 342)	Moderate (*N* = 278)	Severe (*N* = 47)	Death (*N* = 17)
Opioids	47 (13.7%)	32 (11.5%)	5 (10.6%)	9 (52.9%)
Antihypertensives	34 (9.9%)	26 (9.4%)	6 (12.8%)	2 (11.8%)
Antidepressants	26 (7.6%)	24 (8.6%)	1 (2.1%)	1 (5.9%)
Anticonvulsants	26 (7.6%)	19 (6.8%)	0 (0.0%)	1 (5.9%)
Antibiotics	19 (5.5%)	17 (6.1%)	2 (4.3%)	0 (0.0%)
Vaccines	18 (5.3%)	16 (5.8%)	2 (4.3%)	0 (0.0%)
Antipsychotics	15 (4.4%)	13 (4.7%)	2 (4.3%)	0 (0.0%)
Antidiabetics	8 (2.3%)	7 (2.5%)	1 (2.1%)	0 (0.0%)
Antithyroids	8 (2.3%)	8 (2.9%)	0 (0.0%)	0 (0.0%)
Corticosteroids	7 (2.0%)	5 (1.8%)	2 (4.3%)	0 (0.0%)
Anticoagulants	6 (1.8%)	6 (2.2%)	0 (0.0%)	0 (0.0%)
Eye treatments	6 (1.8%)	6 (2.2%)	0 (0.0%)	0 (0.0%)
Proton-pump inhibitors (PPI)	6 (1.8%)	5 (1.8%)	1 (2.1%)	0 (0.0%)
Benzodiazepines	5 (1.5%)	2 (0.7%)	2 (4.3%)	1 (5.9%)
Diuretics	5 (1.5%)	5 (1.8%)	0 (0.0%)	0 (0.0%)
Insulin	5 (1.5%)	4 (1.4%)	1 (2.1%)	0 (0.0%)
Antiarrhythmics	4 (1.2%)	3 (1.1%)	1 (2.1%)	0 (0.0%)
Hormones	4 (1.2%)	2 (0.7%)	2 (4.3%)	0 (0.0%)
Antiplatelets	3 (0.9%)	2 (0.7%)	1 (2.1%)	0 (0.0%)
Immunosuppressants	3 (0.9%)	1 (0.4%)	2 (4.3%)	0 (0.0%)

## 4 Discussion

During just over 3 years between 2018 and 2021, 31,768 reports of medication-related events were captured using the AIMS Pharmapod platform in Ontario, of which over one-third were near misses. A rapid uptake in reporting during the first quarter of 2019 was observed coinciding with the expansion of the AIMS Program across all Ontario community pharmacies, however the number of reports made by each pharmacy was highly skewed. Furthermore, almost one-quarter of events were identified by the involved patient or their agent, highlighting the importance of patient involvement in the medication management process which has been shown to aid in a reduction of errors that reach the patient.^25^^,^^26^ Important areas to target in the pursuit of improving patient safety were identified, as more than half of all events were attributed to either staffing issues or a lack of adequate quality control systems.

These findings build on those of previous studies highlighting the impact of implementing an anonymized, mandated medication error reporting system within healthcare settings.^5^^,^^27^ Following the initial recruitment period of Ontario community pharmacies, the number of events reported to the AIMS Program increased substantially with a quarterly high of over 3600 events. Comparable trends in reporting uptake have been observed in Nova Scotia, Canada where a mandated anonymized medication error reporting system has been in place since 2010.^5^ An analysis of data collected over 7 years found that on average, over 14,000 events were reported each year, with all community pharmacies located in the province reporting at least once over the study period.^5^ An assessment surrounding the uptake of medication error reporting in Nova Scotia found that reporting compliance among community pharmacies significantly improved over time, however, variation based on factors such as pharmacy type (e.g., corporate, independent) was observed.^28^ Although reporting compliance among Ontario community pharmacies increased over the study period, close to half of all pharmacies did not submit any reports to the AIMS Program. The novelty of the program as compared to the more mature surveillance system in Nova Scotia may explain this observed difference, however we anticipate that compliance will continue to grow over time. Continued emphasis on the importance of medication incident and near miss reporting and dissemination of the learnings identified through analysis of the reports collected through the AIMS Program may aid in stimulating further reporting across all community pharmacies in Ontario.

The high degree of reporting unevenness by pharmacies observed in this study is also important, as it suggests that there is a clustering of reported events among a small proportion of community pharmacies. The number of reports per pharmacy ranged dramatically (from 1 to 1280), suggesting that results may be representative of the types of events occurring in higher reporting pharmacies rather than events occurring across all pharmacies provincially. The large variation in reporting by pharmacy is in line with the distribution of reporting in Nova Scotia, which ranged from 1 to 2806 events per community pharmacy.^5^ More research is needed to better understand the pharmacy characteristics and circumstances leading to some pharmacies reporting in high volumes as compared to others, such as comfort in reporting to the AIMS Program or the increased occurrence of higher severity events.

Our analysis of the reported factors and sub-factors related to the occurrence of each event provides important insights into potential areas where strategies to prevent future events can be implemented. Environmental staffing problems and associated sub-factors of interruptions, high volume dispensing and heavy workload were selected in 30% of events, respectively. This provides preliminary evidence that a significant proportion of events may be related to staffing issues. By ensuring that all pharmacies are adequately staffed to handle high workplace demands and allowing pharmacy professionals to take the time required to follow set procedures, medication errors may be prevented. Additionally, both a lack of quality control systems and staff education with related sub-factors of performing independent checks for high-risk drugs, failure to follow established processes, and equipment control checks contributed to over one-third of all events. Indeed, it is critical that all pharmacy professionals have adequate training, opportunities for continued self-development and the resources required to perform their duties. This coupled with strong quality control systems in place to catch errors before they reach the patient has the potential to help reduce errors. Targeting these forementioned areas may also aid in increased reporting to medication error surveillance systems by helping to address factors frequently cited as barriers to reporting medication errors. This includes time constraints, high workload, pharmacy location, fear of litigation, issues with the reporting platform usability and a perceived lack of feedback or positive change resulting from error reports.^29^^,^^30^

Our findings also highlight several potential important learnings surrounding the types of medications most involved in higher severity events. The medications commonly leading to events overall and those resulting in patient harm aligned closely with commonly prescribed medications, such as antihypertensives, opioids, and antidepressants. Among incidents resulting in patient death, over half involved opioids, reflecting the increased potential for patient harm following opioid-related events. Extra caution should be exerted by pharmacy professionals when dispensing medications that have a higher potential to cause significant patient harm, such as ensuring that the responsible pharmacist double checks that prescriptions are accurately filled prior to signing off on medications to be dispensed. Interestingly, over 6% of all reported events resulted in some degree of patient harm, compared to other reporting systems where only 1% of events were associated with patient harm.^5^^,^^31^ In contrast, a study conducted in Denmark found that of all events that reached the patient, over 70% were classified as having a potentially moderate level of severity if left uncorrected.^32^ Differences in the proportion of events resulting in patient harm across reporting systems may be due to differences in the harm assessment process (e.g., based on the pharmacy professional's personal assessment or actual patient harm), the proportion of reported near misses compared to incidents or reflective of the types of events reported by high volume reporting pharmacies. Overall, this study highlights the importance of establishing mandatory reporting systems for the collection of medication incidents and near misses and provides areas for improvement in prescribing pathways and processes across community pharmacies in Ontario.

Strengths of this study include the analysis of a large number of medication-related event reports collected from community pharmacies across a variety of settings in Ontario using a standardized reporting platform. Nonetheless, this study also had several limitations. First, due to the lack of data on total number of prescriptions dispensed over the study period error rates could not be calculated. Similarly, the volume with which medications are dispensed was not controlled for due to the descriptive nature of this analysis. Therefore, medications that appeared to be most frequently involved in events are strongly correlated with the medications that are dispensed at the highest volumes in pharmacies. Future studies may wish to control for this, to identify medications disproportionately involved in events. Second, as with most surveillance systems, despite the mandatory nature of reporting, the events captured in this system are likely an underestimate of the true prevalence of events.^33^^,^^34^ However, it has previously been shown that medication errors resulting in moderate to severe patient harm are more likely to be prioritized, and therefore captured through mandated reporting systems as compared to events without resultant patient harm.^19^^,^^35^^,^^36^ This may in part explain the greater proportion of events with reported patient harm captured through the AIMS Program as compared to other reporting systems. Third, the higher proportion of reports from a relatively small number of pharmacies has the potential to bias the findings towards the types of events commonly encountered by these pharmacies. Furthermore, over 40% of community pharmacies in Ontario did not submit any reports over the study period, and as such these results may not be generalizable to all community pharmacies in Ontario. Future investigation into the barriers to reporting such as time constraints and high workload may aid in continuing to stimulate reporting across all pharmacies in Ontario. Fourth, due to the inherent nature of the reporting system, this data was derived from unvalidated self-reports which may lack accuracy and completeness, leading to a potential misclassification of reported events. Finally, most mandatory reporting fields included “other” categories that were selected in a relatively high proportion of events (i.e., 32.9% of all contributory factors). In some cases, these fields are accompanied by free-text fields where additional information can be entered. Without access to this information, conclusions cannot be made about the types of events and causal factors associated with these categories. Therefore, future research is needed to determine the impact of these missing data on the observed trends in reporting.

## 5 Conclusions

Since implementation of the AIMS Program in Ontario, the number of reporting community pharmacies has substantially increased. Overall, these findings provide important insights into the level of engagement by community pharmacy teams with the AIMS Program and initial learnings such as the types of medications and contributory factors involved in reported events that can help inform recommendations for pharmacy practice and improvement of the program. However, as only 60% of all community pharmacies in Ontario reported at least 1 event, continued education on the importance of the program and obligation to report all events may aid in stimulating further reporting. Increases in both the frequency and the quality of reports made by pharmacy professionals across Ontario will aid in the continued identification of the circumstances surrounding medication errors and development of strategies and resources to help prevent future events.

## Funding

This work was supported by the 10.13039/100014469Ontario College of Pharmacists. However, the opinions, results and statements expressed herein are solely those of the authors and are independent from the funding source. No endorsement is intended or should be inferred. T. Gomes is supported by a Tier 2 Canada Research Chair.

## CRediT authorship contribution statement

**Shaleesa Ledlie:** Conceptualization, Methodology, Investigation, Writing – original draft, Writing – review & editing. **Tara Gomes:** Conceptualization, Methodology, Supervision, Writing – review & editing. **Lisa Dolovich:** Conceptualization, Writing – review & editing. **Chantelle Bailey:** Conceptualization, Writing – review & editing. **Saira Lallani:** Conceptualization, Writing – review & editing. **Delia Sinclair Frigault:** Conceptualization, Writing – review & editing. **Mina Tadrous:** Conceptualization, Methodology, Supervision, Writing – review & editing.

## Declaration of Competing Interest

The authors declare that they have no known competing financial interests or personal relationships that could have appeared to influence the work reported in this paper.

## Appendix A Supplementary data

AIMS Program electronic reporting form fields Image 1

## References

[1] Institute for Safe Medication Practices Canada (ISMP) (2022). Definition of Terms. https://www.ismp-canada.org/definitions.htm.

[2] Hong K., Hong Y.D., Cooke C.E. (2019). Medication errors in community pharmacies: the need for commitment, transparency, and research. Res Social Adm Pharm.

[3] Institute for Safe Medications Practice Canada (2020). Medications Most Frequently Reported in Harm Incidents over the Past 5 Years (2015–2020). https://ismpcanada.ca/wp-content/uploads/ISMPCSB2020-i11-Medications-Reported-Harm.pdf.

[4] Lynskey D., Haigh S., Patel N., Macadam A. (2007). Medication errors in community pharmacy: an investigation into the types and potential causes. Int J Pharm Pract.

[5] Boucher A., Ho C., MacKinnon N. (2018). Quality-related events reported by community pharmacies in Nova Scotia over a 7-year period: a descriptive analysis. Can Med Assoc Open Access J.

[6] Canadian Patient Safety Institute Medication Without Harm - Canada's Contribution to a Global Effort to Reduce Medication Errors. https://www.patientsafetyinstitute.ca/en/NewsAlerts/News/pages/medication-without-harm-2018-09-14.aspx.

[7] Kane Larson C.L. (2019). Potential Cost Savings and Reduction of Medication Errors Due to Implementation of Computerized Provider Order Entry and Bar–Coded Medication Administration in the Fraser Health Authority. Univ Br C Med J.

[8] Hohl C.M., Nosyk B., Kuramoto L. (2011). Outcomes of emergency department patients presenting with adverse drug events. Ann Emerg Med.

[9] World Health Organization (WHO) (2017).

[10] Institute for Safe Medication Practices Canada (2022). About Us. https://www.ismp-canada.org/aboutus.htm.

[11] Allen Kachalia M.D., J. (2013). Improving patient safety through transparency. N Engl J Med.

[12] Tsao K., Browne M. (2015).

[13] Ontario College of Pharmacists (OCP) (2022). About the College. https://www.ocpinfo.com/about/.

[14] National Association of Pharmacy Regulatory Authorities (NAPRA) (2022). National Statistics. https://www.napra.ca/national-statistics.

[15] Ontario College of Pharmacists (OCP) (2022). Glossary of Terms. https://www.ocpinfo.com/protecting-the-public/about-find-a-pharmacy-or-pharmacy-professional/glossary/.

[16] Ontario College of Pharmacists (OCP) (2016). AIMS Backgrounder. https://www.ocpinfo.com/wp-content/uploads/documents/AIMS-medication-safety-backgrounder.pdf.

[17] Ontario College of Pharmacists (OCP) (2022). Supplemental Standard of Practice: Mandatory Standardized Medication Safety Program in Ontario Pharmacies. https://www.ocpinfo.com/library/consultations/download/Supplemental%20Standard%20of%20Practice.pdf.

[18] Campbell P.J., Patel M., Martin J.R. (2018). Systematic review and meta-analysis of community pharmacy error rates in the USA: 1993–2015. BMJ Open Qual.

[19] Donaldson M.S., Corrigan J.M., Kohn L.T. (2000).

[20] Nosek R.A., McMeekin J., Rake G.W. (2005). Advances in patient safety: from research to implementation.

[21] Boyle T.A., MacKinnon N.J., Mahaffey T., Duggan K., Dow N. (2012). Challenges of standardized continuous quality improvement programs in community pharmacies: the case of SafetyNET-Rx. Res Social Adm Pharm.

[22] Von Elm E., Altman D.G., Egger M. (2007). The strengthening the reporting of observational studies in epidemiology (STROBE) statement: guidelines for reporting observational studies. Ann Intern Med.

[23] Ontario College of Pharmacists (OCP) (2022). AIMS Program E-Training. https://www.ocpinfo.com/regulations-standards/aims-assurance-and-improvement-in-medication-safety/aims-program-e-training/.

[24] Berndt D.J., Fisher J.W., Rajendrababu R.V., Studnicki J. (2003). 36th Annual Hawaii International Conference on System Sciences.

[25] Armstrong N., Herbert G., Aveling E.L., Dixon-Woods M., Martin G. (2013). Optimizing patient involvement in quality improvement. Health Expect.

[26] Vaismoradi M., Jordan S., Kangasniemi M. (2015). Patient participation in patient safety and nursing input–a systematic review. J Clin Nurs.

[27] Rishoej R.M., Almarsdóttir A.B., Christesen H.T., Hallas J., Kjeldsen L.J. (2017). Medication errors in pediatric inpatients: a study based on a national mandatory reporting system. Eur J Pediatr.

[28] Boyle T.A., Bishop A.C., Overmars C. (2015). Uptake of quality-related event standards of practice by community pharmacies. J Pharm Pract.

[29] (2022). AIMS Engagement Survey Results.

[30] Williams S.D., Phipps D.L., Ashcroft D.M. (2013). Understanding the attitudes of hospital pharmacists to reporting medication incidents: a qualitative study. Res Social Adm Pharm.

[31] MacKinnon N.J., Boucher A., Barker J., Ho C. (2020). Drugs associated with quality-related events reported by community pharmacies in Nova Scotia, Canada. BMJ Open Qual.

[32] Knudsen P., Herborg H., Mortensen A., Knudsen M., Hellebek A. (2007). Preventing medication errors in community pharmacy: frequency and seriousness of medication errors. BMJ Qual Saf.

[33] Hazell L., Shakir S.A. (2006). Under-reporting of adverse drug reactions. Drug Saf.

[34] Mahajan R. (2011). Medication errors: can we prevent them?. Br J Anaesth.

[35] Sarvadikar A., Prescott G., Williams D. (2010). Attitudes to reporting medication error among differing healthcare professionals. Eur J Clin Pharmacol.

[36] Gavaza P., Brown C.M., Lawson K.A., Rascati K.L., Wilson J.P., Steinhardt M. (2011). Influence of attitudes on pharmacists’ intention to report serious adverse drug events to the Food and Drug Administration. Br J Clin Pharmacol.

