# Supplementary material for: Medication errors in community pharmacies: Evaluation of a standardized safety program
Source: Explor Res Clin Soc Pharm. 2022 Dec 21;9:100218. doi: 10.1016/j.rcsop.2022.100218 (PMC9827048; doi:10.1016/j.rcsop.2022.100218)
Supplement: Supplementary file 1 — AIMS Program electronic reporting form fields [file mmc1.docx]

**Appendix A: AIMS Program electronic reporting form fields**

| **Field** | **Categorical Options** |
| --- | --- |
| Pharmacy identification number | N/A |
| Patient month and year of birth | N/A |
| Patient gender | Female |
|  | Male |
|  | Other |
| Error type | Incident |
|  | Near miss |
| Event category | Drug therapy monitoring problem |
|  | Duplication of therapy |
|  | Incorrect concentration |
|  | Incorrect dosage form |
|  | Incorrect drug |
|  | Incorrect duration |
|  | Incorrect frequency |
|  | Incorrect patient |
|  | Incorrect quantity |
|  | Incorrect route administration |
|  | Incorrect storage |
|  | Medication inappropriately discontinued |
|  | Medication incorrect label |
|  | Medication incorrect prescriber |
|  | Medication omitted medication dose |
|  | Other |
|  | Passed expiry date |
|  | Prescribing error |
|  | Professional services incident |
| Severity assessment of patient harm | None |
|  | Mild |
|  | Moderate |
|  | Severe |
|  | Death |
|  | Unknown |
| Date the event occurred | N/A |
| Time the event occurred | N/A |
| Individual who identified the event | Nurse |
|  | Other healthcare provider |
|  | Patient |
|  | Patient agent |
|  | Pharmacist |
|  | Pharmacy assistant |
|  | Pharmacy student |
|  | Pharmacy technician |
|  | Prescriber |
|  | Social worker |
| Product prescribed | N/A |
| Product dispensed | N/A |
| Patient indication | N/A |
| Event stage | Administration |
|  | Communication |
|  | Delivery |
|  | Dispensing |
|  | Order entry |
|  | Other |
|  | Prescribing |
|  | Product selection |
|  | Storage |
|  | Supply |
| Contributory event factors | Drug-related issues |
|  | Environmental staffing problem |
|  | Lack of quality control systems |
|  | Lack of staff education |
|  | Miscommunication of drug order |
|  | Other contributory factors |
|  | Patient caregiver education problem |
|  | Patient education problem |
|  | Patient information missing |
| Contributory event sub-factors | Additional clinical references available were not reviewed at the time |
|  | Age |
|  | Alike packaging |
|  | Allergies |
|  | Ambiguous |
|  | Appropriate information not provided to patient |
|  | Backorder medication |
|  | Clutter |
|  | Competence training factor |
|  | Default |
|  | Difficulty accessing prescriber |
|  | Employee safety |
|  | Equipment control checks |
|  | Failure to follow established process |
|  | Fatigue |
|  | Heavy workload |
|  | High volume dispensing |
|  | Higher than normal volume dispensing period |
|  | Illegible |
|  | Inadequate lighting |
|  | Incomplete |
|  | Independent checks high alert risk patient drugs |
|  | Indication |
|  | Inefficient ineffective workflow |
|  | Inefficient workflow |
|  | Interruptions |
|  | Interruptions due to OTC consults |
|  | Interruptions due to phone calls |
|  | Interruptions due to professional services flu shots or medication reviews |
|  | Interruptions resulted in longer prescription processing time |
|  | Interruptions resulted in manual steps to speed up process |
|  | Intimidation faulty interaction |
|  | Lab values |
|  | Lack of feedback |
|  | Lack of orientation process |
|  | Lighting |
|  | Light sensitive medication |
|  | Location |
|  | Look and sound alike names |
|  | Lower than normal volume dispensing period |
|  | Medication not covered |
|  | Misheard orders |
|  | Misunderstood orders |
|  | Multiple concentrations |
|  | New unfamiliar drug |
|  | No or limited supervision |
|  | Noise |
|  | Other contributory factors |
|  | Outdated references |
|  | Patient identity |
|  | Pharmacist did not ensure patient understanding |
|  | Pregnancy |
|  | Prescribed dosage not available |
|  | Process-related established SOPs |
|  | Product-related issue |
|  | Quieter than normal |
|  | Renal liver impairment |
|  | Scheduled staff insufficient |
|  | Staff changeover or breaks |
|  | Staff fatigue |
|  | Staffing deficiencies |
|  | Supervision deficiency |
|  | System-related pharmacy management system |
|  | System-related pharmacy tools or technology |
|  | Taking on multiple tasks/activities |
|  | Time constraints to meet a cut off time |
|  | Unclear absent labelling |
|  | Unfamiliar process |
|  | Unplanned or non-scheduled non dispensing activities |
|  | Unplanned or unscheduled dispensing activities |
|  | Vital signs |
|  | Weight |
